# Death of Dr. Houghton

**Published:** 1845-12

**Authors:** 


### DEATH OF DR. HOUGHTON.

By the newspapers, we have learned, with deep regret, that Dr. Douglass Houghton, State Geologist of Michigan, was lately drowned in Lake Superior, on the shores of which he had been for sometime engaged in a mineral survey, for the general government. In our next number we hope to be able to give a sketch of the labors and character of this indefatigable naturalist.

